# Obesity Health Screening in Japanese School-Aged Children and Adolescents

**DOI:** 10.7759/cureus.100766

**Published:** 2026-01-04

**Authors:** Hisako Tanaka, Naho Morisaki, Shohei Harada, Kevin Y Urayama

**Affiliations:** 1 Department of Social Medicine, National Center for Child Health and Development, Tokyo, JPN; 2 Department of Medical Affairs, Akitsu Ryoikuen, Tendokai Social Welfare Corporation, Tokyo, JPN

**Keywords:** chronic diseases, overweight, pediatric obesity, schools, screening

## Abstract

Background: Addressing obesity in childhood may alleviate disease risk in later life. Using real-world data from a school health obesity screening program, we examined the percentage of overweight (POW) by sex, grade, blood test results, and lifestyle factors to inform programmatic improvements.

Materials and methods: We analyzed data from lifestyle-related disease prevention examinations conducted in Setagaya Ward for children and adolescents between 2015 and 2019 (n = 571). Schoolchildren were categorized into three groups based on POW at the school health exam and change in POW at follow-up. Descriptive analyses were performed to identify factors associated with these outcomes.

Results: Comparing the percentage of students in the decreased POW group, 72.3% (115/159) of junior high school students experienced a decrease, compared with 49.5% (204/412) of elementary school students. There was a tendency for no change in POW or increased POW among schoolchildren with higher aspartate aminotransferase, alanine aminotransferase, and total cholesterol. Sleep time tended to be longer in the lower POW group and shorter in the POW 50 group. Television viewing time on weekdays and holidays was shorter in the lower POW groups.

Conclusions: Higher obesity percentages were observed among boys and among children in higher grades. Given the associations with lifestyle factors as well, it may be essential to integrate more targeted health guidance in the screening program.

## Introduction

Childhood obesity is a public health issue of concern in many countries, including Japan. In 2022, the proportion of upper elementary students with a percentage of overweight (POW) over 20% was about 14% among boys and 10% among girls [1]. Obesity continues to be a growing problem in light of the COVID-19 pandemic in 2020. It had a significant impact on the lives of children, such as the increase in obesity as a result of reduced energy consumption due to restrictions on going out [2]. There is also a tendency for overweight and obesity during childhood to continue into adulthood and influence the risk of various medical conditions [3]. More than half of all causes of death in Japan have been estimated to be due to lifestyle-related diseases [4].

In children as well, obesity-associated lifestyle-related diseases and their complications have been on the rise [3]. As a result, many municipalities across Japan have implemented screening among school-age children for lifestyle-related diseases and their risk factors and provide medical intervention when deemed necessary. However, there is variation across municipalities in screening methods, criteria for identifying who to provide intervention to, and the intervention method used [5]. This inconsistency is partly due to the lack of longitudinal evidence regarding program-related outcomes.

Setagaya Ward is one of the most populated and resource-rich municipalities in Tokyo. It has provided an Obesity Health Screening (currently called “Screening for Lifestyle-related Diseases in Schoolchildren”) since 1978. Analysis of screening results for the early years of this program, prior to 1998, has shown an increasing trend in the incidence of obese children, consistent with national data trends [1]. We previously reported descriptive observations of secular variation in obesity between the years 2009 and 2018 [6]. A report from Takaoka City, Toyama Prefecture, showed a positive effect of continuing preventive screening for pediatric lifestyle-related diseases [7]. However, nationwide implementation has not occurred, and additional evidence on the effects of such programs can help inform the development of a cost-effective examination system.

In this study, we examined individual-level real-world data from the annual school health checkup and “Screening for Lifestyle-related Diseases in Schoolchildren” (hereinafter referred to as “Obesity Health Screening”) implemented in Setagaya Ward from 2015 to 2019. The objectives were to conduct a descriptive evaluation of unique data from an active obesity screening program to understand the cross-sectional profile of participants by overweight/obesity status and their short-term longitudinal change. We examine associations between POW status and blood test parameters, lifestyle factors, and demographic characteristics. These results are expected to potentially inform future strategies for optimizing program outcomes for children facing issues of overweight and obesity.

## Materials and methods

Study context and population

As described in a previous report [6], all Japanese students, from kindergarten through university, undergo the annual school health checkup administered by their school. These are conducted in accordance with the provisions of the School Education Law and the School Health and Safety Law of Japan. This includes various anthropometric measurements, urinalysis, assessment of hearing and visual acuity, and the presence or absence of disease or abnormality, along with a child health questionnaire completed by a parent or guardian (up to high school age). This study examined a subset of students participating in the Obesity Health Screening program in Setagaya Ward, the largest and most populous of the 62 municipalities in Tokyo. As of 2023, the area is home to approximately 74,300 children and adolescents (ages 5-14) [8]. According to the 2023 School Basic Survey, among 61 public elementary schools and 29 public junior high schools, there were about 38,300 [9] and 11,900 students enrolled, respectively [10]. The National Center for Child Health and Development Ethics Review Committee issued approval 1854.

Obesity measurement and screening

In Japan, the POW measure is used more commonly than the BMI-for-age percentile and is calculated from measured weight and standard weight-for-height as follows [11,12].

$\text{POW (\%)} = 100 \left( \frac{W_{\mathrm{measured}} - W_{\mathrm{standard}}}{W_{\mathrm{standard}}} \right)$

The standard weight is age- and sex-specific and was derived from data from the Infant and Child Physical Development Survey and the School Health Statistics Survey Report in 2000 (Ministry of Education, Culture, Sports, Science and Technology) [13]. A child with a POW ≥20% (POW 20) is considered mildly obese, ≥30% (POW 30) as moderately obese, and ≥50% (POW 50) as severely obese. Schoolchildren with a POW 30 at the school health exam falling under one of the following conditions were targeted for follow-up participation in the Obesity Health Screening: (1) in the second or fourth grade of elementary school or first year of junior high school, (2) those receiving a “re-examination after one year” or who “require a detailed examination” and have not been screened at a professional medical organization, or (3) those outside of the targeted grades but recommended by the school to receive screening. For those who underwent consecutive screenings, data from the first screening were retained, and data from subsequent screenings were excluded.

**Figure 1:**
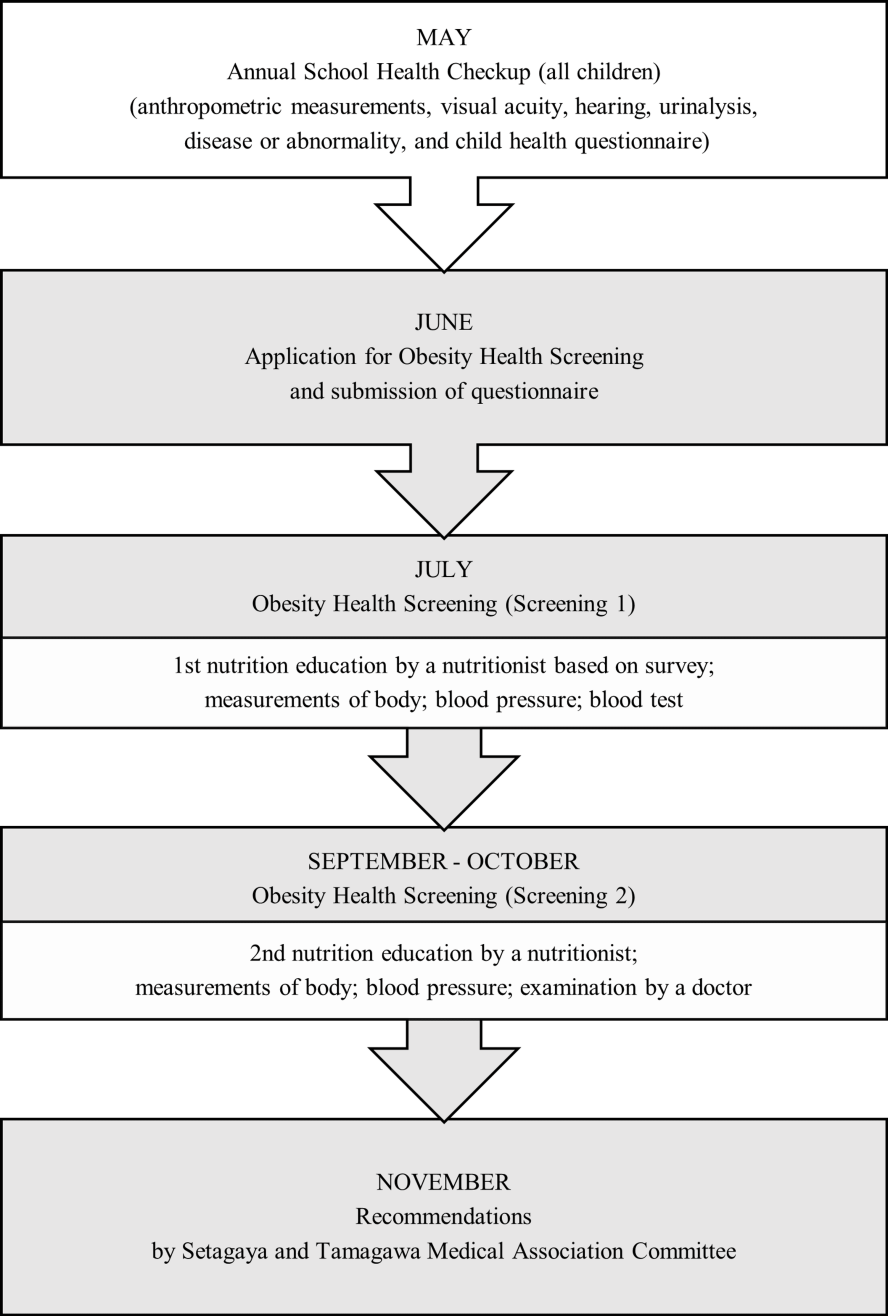
Description of flow of the annual school health check-up and the Obesity Health Screening (screenings 1 and 2) administered in Setagaya Ward, Tokyo

Figure 1 shows the flow of student inclusion into the Obesity Health Screening. Schoolchildren with POW 30 at the school health exam, administered annually in May, were notified and allowed to participate in the Obesity Health Screening. Participants submit a questionnaire regarding their diet and lifestyle habits in June at the same time as their application. The questionnaire consisted of three parts: (1) a one-week assessment of eating habits, (2) a three-day dietary record, and (3) an assessment of lifestyle habits. The dietary and lifestyle questionnaire is used in two nutrition education sessions provided to parents and children. These nutrition education sessions are conducted by Showa Women's University, and registered dietitians who attended the pre-information session will provide one-on-one nutritional guidance based on the results of a questionnaire designated by Setagaya Ward. The first session is held in July to set effort goals (hereinafter referred to as “screening 1”), and the second session is held in September to October to confirm goal achievement and provide advice for the future (hereinafter referred to as “screening 2”). At screening 1 in July, measurements are taken, including body size (height, weight, abdominal circumference, and percentage of body fat), blood pressure (measured up to three times, if elevated), and a blood test. Blood tests were conducted at the school doctor's medical facility; subjects had blood drawn between 8 and 9 a.m. on an empty stomach without eating breakfast. Test items are aspartate aminotransferase (AST), alanine aminotransferase (ALT), total cholesterol (TC), triacylglycerol (TG), high-density lipoprotein cholesterol (HDL-C), blood glucose (BS), hemoglobin A1c (HbA1c), immunoreactive insulin (IRI), and uric acid (UA), as designated by Setagaya Ward. At screening 2, approximately two months later, participants of screening 1 obtain a second round of nutritional consultation, body size and blood pressure measurements, and feedback from a physician on the blood test results.

Variables

This study used five-year screening data from Setagaya Ward, from 2015 to 2019. The survey items included sex, grade, height, weight, POW, body fat, abdominal circumference, blood pressure, blood test, medical examination results, and lifestyle characteristics. Blood test items included measures for AST, ALT, TC, TG, HDL-C, BS, HbA1c, IRI, and UA. Additionally, clinical cut points for TC, TG, and HDL in children were considered as follows [14]: TC: acceptable (mg/dL); <170, borderline; 170 to 199, high; ≥200, TG (0 to 9 years): acceptable (mg/dL); <75, borderline; 75 to 99, high; ≥100, TG (10 to 19 years): acceptable (mg/dL); <90, borderline; 90 to 129, high; ≥130, HDL-C: acceptable (mg/dL); >45, borderline; 40 to 45, high; <40. The lifestyle questionnaire included items on birth weight, sleep duration, television viewing time, exercise habits, and eating habits.

**Figure 2:**
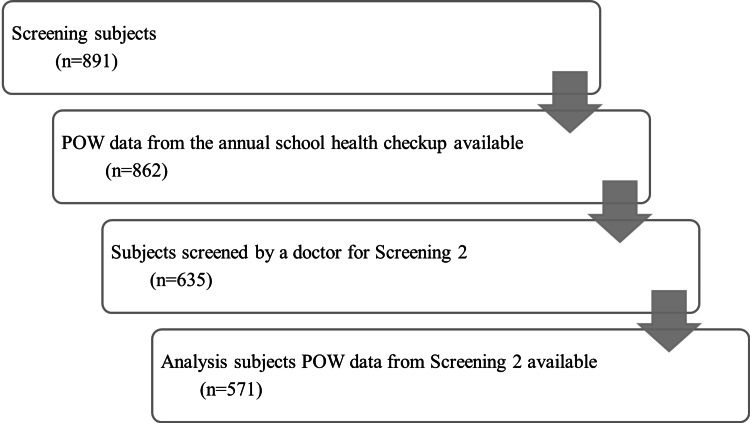
Participant flow POW: percentage of overweight

Data were available for 862 children who underwent medical examinations from 2015 to 2019; 571 participated in screening 2 and were included in this analysis (Figure 2). Among 571 children, 555 had data on blood test results, and 295 had data on lifestyle-related factors.

Statistical analysis

Analyses utilized the following three sub-populations: (1) 571 children who had data at school exam and screening 2, (2) 555 children who had results from a blood test taken during the screening exam, and (3) 295 children who submitted a lifestyle-related questionnaire during the screening exam. Children who participated in the examination were categorized into three groups based on POW at the school health exam of 30-40, 40-50, and >50. Additionally, children were divided into two groups: a “reduced POW” category or a "no change or increased POW” category based on the difference in POW between screening 2 and the school health exam. Children in each category were compared on demographic data, lifestyle-related habits, and blood test results, and descriptive analyses were performed. The chi-square test and Fisher's exact test were used for categorical variables. The Kruskal-Wallis test and the Mann-Whitney U test were used to compare the medians across the POW categories, and one-way analysis of variance and t-test were used to compare mean values. Multivariable analyses were performed using logistic regression to evaluate the association between each covariate and POW at the school health exam (POW 30-40 vs >40) and the change in POW from screening 1 to screening 2. We performed logistic regression with the dependent variables: school health checkup obesity levels or changes in obesity levels, and the independent variables: sex, grade, AST, ALT, BS, HbA1c, IRI, UA, TC, TG, and HDL-c. Additionally, a similar logistic regression analysis was conducted using the independent variables as gender, grade, father's BMI, mother's BMI, child's birth weight, sleeping time, TV viewing time, and snack habits. A two-sided p-value of 0.05 was considered statistically significant.

## Results

Characteristics of basic data for each classification

**Figure 3:**
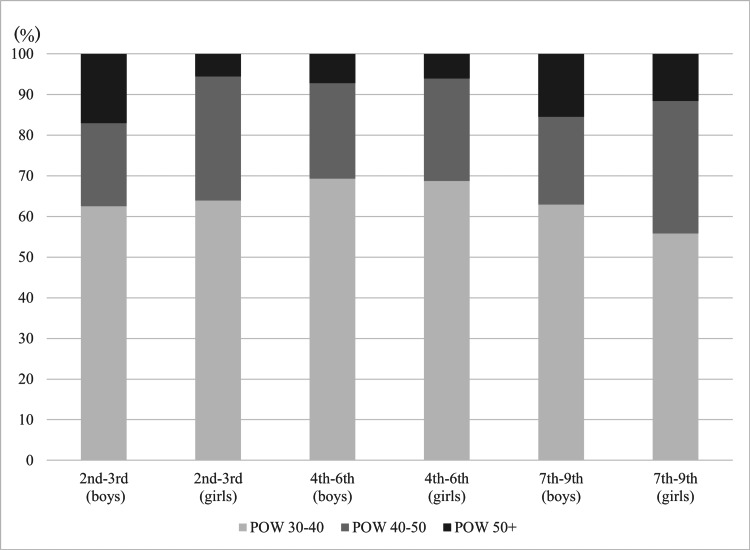
Distribution of POW by sex and grade level among participants of the Obesity Health Screening POW: percentage of overweight

A total of 571 children participating in screening 2 were classified by grade and sex according to POW at school exam, and POW 30-40 comprised the majority across all grade and sex categories, ranging from about 55% (7th-9th girls: 24/32) to 70% (7th-9th boys: 106/153) (Figure 3). There was a higher percentage of boys than girls in all POW categories, but it was highest among POW >50 (44, 74.6%) (Table 1). When examining the difference in POW between the school exam and screening 2, about 56% (319/571) experienced a reduction in POW. There was a higher percentage of 7th-9th graders among the reduced POW category compared to the no change or increased POW category (36.1% vs 17.5%).

**Table 1:** Characteristics of schoolchildren participating in the Obesity Health Screening by POW status * statistical significance POW: percentage of overweight, χ²: chi-square test statistic, df: degrees of freedom

Characteristic	Category	Total (n = 571)		POW at school exam								Change in POW (school exam - exam 2)					
				POW -40 (n = 372)		POW 40-50 (n = 140)		POW 50+ (n = 59)		χ2 (df)	P	Reduced POW (n = 319)		No change or increased POW (n = 252)		χ2 (df)	P
		n	%	n	%	n	%	n	%			n	%	n	%		
Sex	Male	357	62.5	234	62.9	79	56.4	44	74.6	5.90 (2)	0.05	197	61.8	160	63.5	0.18 (1)	0.67
	Female	214	37.5	138	37.1	61	43.6	15	25.4			122	38.2	92	36.5		
Grade	2^nd^-3^rd^	160	28.0	101	27.2	40	28.6	19	32.2	7.27 (4)	0.12	75	23.5	85	33.7	24.95 (2)	<0.01*
	4^th^-6^th^	252	44.1	174	46.8	61	43.6	17	28.8			129	40.4	123	48.8		
	7^th^-9^th^	159	27.8	97	26.1	39	27.9	23	39.0			115	36.1	44	17.5		
POW at school exam	POW 40	372	65.1	－	－	－	－	－	－	－	－	210	65.8	162	64.3	0.19 (2)	0.91
	POW 40-50	140	24.5	－	－	－	－	－	－	－	－	76	23.8	64	25.4	－	－
	POW 50+	59	10.3	－	－	－	－	－	－	－	－	33	10.3	26	10.3	－	－

Blood tests and blood pressure

Table 2 shows median blood measurements for select laboratory measures related to obesity and metabolic health across POW at school exam categories. POW at school exams was associated with ALT, HDL-C, IRI, UA, and diastolic pressure (p < 0.05), with healthier median values observed among those in lower POW categories. When comparing children with a reduction in POW to those with no change or increased POW, significant differences were observed in AST, ALT, TC, TG, and IRI (p < 0.01). The directionality of median values among children who experienced a reduction in POW was consistent with a generally improved blood test profile. These associations persisted in multivariable logistic regression analyses (Table 3), even after adjusting for sex and grade level (p < 0.05). When examining clinically relevant categories for TC and TG, higher odds of no change or increased POW were associated with borderline or high categories compared to those with acceptable levels. Additionally, in this study, the average serum cholesterol levels (TC, HDL-C, TG) were lowest in the 7th-9th graders and highest for TC and TG in the 4th-6th graders.

**Table 2:** Summary of blood test results by POW at school exam categories and changes in POW POW: percentage of overweight, AST: aspartate aminotransferase, ALT: alanine aminotransferase, TC: total cholesterol, TG: triacylglycerol, HDL-C: high-density lipoprotein cholesterol, BS: blood sugar, HbA1c: hemoglobin A1c, IRI: immunoreactive insulin, UA: uric acid

	Total		POW at school exam							Change in POW (school exam - exam 2)				
			POW -40		POW 40-50		POW 50+		P	Reduced POW		No change or increased POW		P
	n	Median (min-max)	n	Median (min-max)	n	Median (min-max)	n	Median (min-max)		n	Median (min-max)	n	Median (min-max)	
AST	553	25.0 (13-85)	364	25.0 (13-75)	136	24.5 (14-58)	53	26.0 (13-85)	0.06	311	24.0 (13-62)	242	26.0 (14-85)	0.00
ALT	553	19 (6-209)	364	18 (6-113)	136	20 (7-106)	53	26 (10-209)	0.00	311	17 (6-98)	242	22 (9-209)	0.00
TC	553	177 (55-298)	364	178 (111-298)	136	176 (55-291)	53	169 (110-214)	0.15	311	170 (55-272)	242	182.5 (105-298)	0.00
TG	553	81.0 (20-477)	364	78.0 (21-450)	136	85.0 (20-477)	53	96.0 (23-262)	0.14	311	75.0 (21-436)	242	91.5 (20-477)	0.00
HDL-C	553	54.0 (28-91)	364	54.5 (32-91)	136	53.5 (36-85)	53	48.0 (28-78)	0.01	311	54.0 (28-91)	242	54.0 (30-89)	0.99
BS	553	87 (66-140)	363	87 (71-140)	137	85 (66-106)	53	87 (69-108)	0.17	311	87 (69-110)	242	87 (66-140)	0.92
HbA1c	553	5.3 (4.5-6)	363	5.3 (4.5-5.9)	137	5.3 (4.8-5.8)	53	5.3 (4.8-6)	0.27	311	5.3 (4.5-6)	242	5.3 (4.8-5.8)	0.97
IRI	552	9.8 (0.5-137.5)	363	9.2 (1.5-137.5)	136	11.1 (1.5-69.4)	53	14.3 (0.5-128.6)	0.00	311	9.2 (0.5-137.5)	241	11.0 (1.5-128.6)	0.00
UA	553	5.1 (1.9-9.5)	364	5.0 (1.9-8.7)	136	5.3 (2.7-8.9)	53	5.6 (3.4-9.5)	0.00	311	5.1 (2.8-8.8)	242	5.0 (1.9-9.5)	0.29
Systolic pressure	551	104.0(46-180)	361	104.0(46-139)	137	104.0(46-180)	53	112.0(48-140)	0.07	309	104.0(46-140)	242	104.0(46-180)	0.86
Diastolic pressure	551	65.0(39-141)	361	64.0(39-141)	137	66(42-129)	53	70.0(42-128)	0.02	309	64.0(40-141)	242	65.0(39-128)	0.68
	n	%	n	%	n	%	n	%		n	%	n	%	
Suspected impaired glucose tolerance	49	8.8	26	7.1	15	10.9	8	14.8	-	26	7.1	23	12.0	-
Suspected liver dysfunction	47	8.5	26	7.1	9	6.6	12	22.2	-	26	7.1	21	11.0	-
Suspected dyslipidemia	180	32.4	115	31.6	46	33.6	19	35.2	-	115	31.6	65	34.0	-
Suspected hypertension	5	0.9	3	0.8	1	0.7	1	1.9	-	3	0.8	2	1.0	-
Severe obesity	59	10.6	26	7.1	21	15.3	12	22.2	-	26	7.1	33	17.3	-
Suspected hyperuricemia	43	7.7	28	7.7	8	5.8	7	13.0	-	28	7.7	15	7.9	-

**Table 3:** Multivariate analysis of POW status associated with sex, grade, and blood test results This table shows odds ratios of "POW at school exam over 40" and "no change or increased POW." TC: acceptable (mg/dL); <170, borderline; 170 to 199, high; ≥200 TG (0 to 9 years): acceptable (mg/dL); <75, borderline; 75 to 99, high; ≥100 TG (10 to 19 years): acceptable (mg/dL); <90, borderline; 90 to 129, high; ≥130 HDL-C: acceptable (mg/dL); >45 (1.2), borderline; 40 to 45 (1 to 1.2), high; <40 (1) * Odds ratios (OR) and 95% confidence intervals (CI) were calculated using multivariable logistic regression, adjusting for sex and grade level. POW: percentage of overweight, AST: aspartate aminotransferase, ALT: alanine aminotransferase, TC: total cholesterol, TG: triacylglycerol, HDL-C: high-density lipoprotein cholesterol, BS: blood sugar, HbA1c: Hemoglobin A1c, IRI: immunoreactive insulin, UA: uric acid

		POW at school exam (-40, 40+)				Change in POW (school exam - screening 2)			
		Unadjusted		Adjusted		Unadjusted		Adjusted	
		OR (95% CI)*	P	OR (96% CI)*	P	OR (95% CI)*	P	OR (96% CI)*	P
Boys (vs girls)		1.07 (0.75-1.54)	0.70	1.11 (0.75-1.66)	0.59	0.95 (0.68-1.35)	0.79	0.89 (0.6-1.32)	0.56
Grade	ES2-3	1.00 (reference)		1.00 (reference)		1.00 (reference)		1.00 (reference)	
	4th-6th	0.79 (0.52-1.20)	0.27	0.58 (0.36-0.93)	0.02	0.83 (0.56-1.25)	0.38	0.66 (0.42-1.05)	0.08
	7th-9th	1.07 (0.67-1.69)	0.78	0.58 (0.32-1.06)	0.08	0.34 (0.21-0.54)	0.00	0.35 (0.19-0.64)	0.00
AST		1.02 (1.00-1.04)	0.06	0.96 (0.92-1.01)	0.16	1.07 (1.04-1.09)	0.00	0.99 (0.94-1.04)	0.65
ALT		1.02 (1.01-1.03)	0.00	1.02 (1.00-1.05)	0.04	1.03 (1.02-1.04)	0.00	1.03 (1.01-1.06)	0.00
BS		0.98 (0.96-1.01)	0.15	0.97 (0.94-1.00)	0.04	1.00 (0.98-1.03)	0.86	1.01 (0.98-1.04)	0.36
HbA1C		1.78 (0.76-4.19)	0.19	1.88 (0.72-4.94)	0.20	1.01 (0.45-2.23)	0.98	0.7 (0.27-1.79)	0.46
IRI		1.02 (1.00-1.03)	0.03	1.01 (0.99-1.03)	0.19	1.01 (1.00-1.03)	0.11	1.01 (0.99-1.02)	0.48
UA		1.33 (1.14-1.55)	0.00	1.25 (1.04-1.50)	0.02	0.91 (0.79-1.05)	0.20	0.9 (0.75-1.08)	0.25
TC	Acceptable	1.00 (reference)		1.00 (reference)		1.00 (reference)		1.00 (reference)	
	Borderline	0.93 (0.63-1.39)	0.74	1.01 (0.65-1.57)	0.95	2.25 (1.52-3.32)	0.00	1.97 (1.27-3.05)	0.00
	High	0.80 (0.50-1.28)	0.35	0.81 (0.47-1.37)	0.43	2.67 (1.69-4.21)	0.00	2.06 (1.24-3.44)	0.01
TG	Acceptable	1.00 (reference)		1.00 (reference)		1.00 (reference)		1.00 (reference)	
	Borderline	1.04 (0.65-1.67)	0.88	0.82 (0.49-1.38)	0.46	1.63 (1.04-2.55)	0.03	1.44 (0.88-2.36)	0.14
	High	1.45 (0.97-2.16)	0.07	1.05 (0.64-1.71)	0.85	2.36 (1.60-3.49)	0.00	1.6 (0.98-2.59)	0.06
HDL-c	Acceptable	1.00 (reference)		1.00 (reference)		1.00 (reference)		1.00 (reference)	
	Borderline	1.84 (1.14-2.98)	0.01	1.6 (0.93-2.74)	0.09	1.33 (0.83-2.14)	0.24	1.44 (0.82-2.52)	0.20
	Low	1.64 (0.82-3.30)	0.16	1.15 (0.53-2.52)	0.72	1.29 (0.65-2.56)	0.47	1.2 (0.54-2.69)	0.65
Systolic pressure		1(0.99-1.01)	0.94	1.00 (0.99-1.02)	0.48	1 (0.99-1.01)	0.92	1 (0.99-1.01)	1.00
Diastolic pressure		1.01(1-1.02)	0.02	1.01 (1.00-1.03)	0.03	1 (0.99-1.01)	0.95	1 (0.99-1.01)	0.92

Lifestyle-related factors captured in the Obesity Health Screening

Table 4 shows the characteristics of a subset of children who completed the lifestyle-related questionnaire, categorized by POW at the school exam and by change in POW. Regarding sleep duration, respondents were asked, "What is your average sleep duration?" with the options being "<7 hours, seven hours, eight hours, nine hours, or more." Regarding television viewing time, they were asked, "How much time do you spend watching television or playing video games?" and were asked about time spent on weekdays and weekends. Regarding physical activity, they were asked, "Do you exercise regularly other than physical education classes?" with the options being "yes" and "no," and those who did were asked about the type of exercise and the time. On average, more than 70% of children had eight hours or less of sleep per night, and about 27% watched >150 minutes of television (television or video games) on weekdays. When examined by POW at school exam categories, children with POW >50 showed the highest percentage of children with <8 hours of sleep (10, 37%), >150 minutes of television on weekdays (13, 50%), and no physical activity (11, 40.7%). Statistically significant associations with change in POW were not observed for these factors. Results of multivariable analyses showed that higher odds of POW at the school exam over 40 were associated with longer durations of television viewing on both weekdays and holidays (Table 5). Children with longer sleep duration >40 had lower odds of a positive outcome on the school exam than children with <8 hours of sleep. The percentage of schoolchildren who slept for eight hours or more was 95.6% (87/91) for second- to third-graders, 88.2% (120/136) for 4th- to 6th-graders, and 53.8% (35/65) for 7th- to 9th-graders.

**Table 4:** Lifestyle-related characteristics and POW status * statistical significance, ^a ^one-way analysis of variance, ^b ^t-test, ^c ^Kruskal-Wallis test, ^d ^Mann-Whitney U test, ^e ^Fisher's exact test POW: percentage of overweight

Characteristic	Category	Total (n = 295)		POW at school exam								Change in POW (school exam - exam 2)					
				POW -40 (n = 189)		POW 40-50 (n = 79)		POW 50+ (n = 27)		χ2 (df)	P	Reduced POW (n = 185)		No change or increased POW (n = 110)		χ2 (df)	P
		n	%	n	%	n	%	n	%			n	%	n	%		
Sex	Male	187	63.4%	122	64.6%	47	59.5%	18	66.7%	0.75 (2)	0.69	73	39.5%	35	31.8%	1.74 (1)	0.19
	Female	108	36.6%	67	35.4%	32	40.5%	9	33.3%			112	60.5%	75	68.2%		
Grade	2^nd^-3^rd^	93	31.5%	59	31.2%	25	31.6%	9	33.3%	2.88 (4)	0.58	56	30.3%	37	33.6%	13.88 (2)	<0.01*
	4^th^-6^th^	136	46.1%	91	48.1%	36	45.6%	9	33.3%			75	40.5%	61	55.5%		
	7^th^-9^th^	66	22.4%	39	20.6%	18	22.8%	9	33.3%			54	29.2%	12	10.9%		
Father age^a,b^	Valid respondents	268	90.8%	174	92.1%	68	86.1%	26	96.3%			168	90.8%	100	90.9%		
	Average (SD)	45.6 (6.0)		45.3 (5.5)		45.7 (7.0)		47.2 (6.2)		-	0.29	46.0 (5.5)		44.8 (6.7)		-	0.12
Father BMI (kg/m2)	Valid respondents	240	81.4%	158	83.6%	59	74.7%	23	85.2%			152	82.2%	88	80.0%		
	<25	125	52.1%	82	51.9%	31	52.5%	12	52.2%	0.01 (2)	1.00	79	52.0%	46	52.3%	<0.01 (1)	0.96
	>25	115	47.9%	76	48.1%	28	47.5%	11	47.8%			73	48.0%	42	47.7%		
Mother age^c,d^	Valid respondents	281	95.3%	181	95.8%	73	92.4%	27	100.0%			176	95.1%	105	95.5%		
	Average (SD)	43.0 (27)		43.0 (27)		43.0 (19)		43.0 (20)		-	0.66	43.0 (22)		43.0 (27)		-	0.19
Mother BMI (kg/m2)	Valid respondents	266	90.2%	176	93.1%	65	82.3%	25	92.6%			167	90.3%	99	90.0%		
	<25	205	77.1%	140	79.5%	46	70.8%	19	76.0%	2.09 (2)	0.35	125	74.9%	80	80.8%	1.25 (1)	0.26
	>25	61	22.9%	36	20.5%	19	29.2%	6	24.0%			42	25.1%	19	19.2%		
Birthweight (kg)^a,b,e^	Valid respondents	247	83.7%	159	84.1%	66	83.5%	22	81.5%			153	82.7%	94	85.5%		
	Average (SD)	3.068 (0.464)		3.087 (0.451)		3.068 (0.496)		2.932 (0.459)		-	0.34	3.044 (0.471)		3.108 (0.451)		-	0.29
	<2.500	25	10.1%	15	9.4%	6	9.1%	4	18.2%	2.39 (4)	0.67	17	11.1%	8	8.5%	1.41 (2)	0.49
	2.500-4.000	217	87.9%	140	88.1%	59	89.4%	18	81.8%			134	87.6%	83	88.3%		
	>4.000	5	2.0%	4	2.5%	1	1.5%	0	0.0%			2	1.3%	3	3.2%		
Hours of sleep	Valid respondents	292	99.0%	187	98.9%	78	98.7%	27	100.0%			184	99.5%	108	98.2%		
	<8	50	17.1%	23	12.3%	17	21.8%	10	37.0%	16.61 (4)	<0.01*	35	19.0%	15	13.9%	1.67 (2)	0.43
	8	158	54.1%	101	54.0%	47	60.3%	10	37.0%			95	51.6%	63	58.3%		
	>9	84	28.8%	63	33.7%	14	17.9%	7	25.9%			54	29.3%	30	27.8%		
Minutes of television (weekdays)	Valid respondents	289	98.0%	186	98.4%	77	97.5%	26	96.3%			183	98.9%	106	96.4%		
	<150	212	73.4%	145	78.0%	54	70.1%	13	50.0%	9.68 (2)	0.01*	137	74.9%	75	70.8%	0.58 (1)	0.45
	>150	77	26.6%	41	22.0%	23	29.9%	13	50.0%			46	25.1%	31	29.2%		
Minutes of television (holidays)^e^	Valid respondents	286	96.9%	183	96.8%	77	97.5%	26	96.3%			179	96.8%	107	97.3%		
	<150	103	36.0%	73	39.9%	26	33.8%	4	15.4%	6.33	0.04*	69	38.5%	34	31.8%	1.33 (1)	0.25
	>150	183	64.0%	110	60.1%	51	66.2%	22	84.6%			110	61.5%	73	68.2%		
Physical activity	Valid respondents	290	98.3%	186	98.4%	77	97.5%	27	100.0%			182	98.4%	108	98.2%		
	Yes	210	72.4%	142	76.3%	52	67.5%	16	59.3%	4.70 (2)	0.10	129	70.9%	81	75.0%	0.58 (1)	0.45
	No	80	27.6%	44	23.7%	25	32.5%	11	40.7%			53	29.1%	27	25.0%		
Frequency of snacking^e^	Valid respondents	291	98.6%	187	98.9%	78	98.7%	26	96.3%			183	98.9%	108	98.2%		
	Every day	180	61.9%	118	63.1%	43	55.1%	19	73.1%	6.68 (4)	0.13	104	56.8%	76	70.4%	5.46 (2)	0.06
	Several times a week	105	36.1%	64	34.2%	35	44.9%	6	23.1%			74	40.4%	31	28.7%		
	Do not eat	6	2.1%	5	2.7%	0	0.0%	1	3.8%			5	2.7%	1	0.9%		

**Table 5:** Analysis of lifestyle and parental characteristics associated with POW status This table shows the odds ratios of "POW at school exam over 40" and "no change or increased POW". * Odds ratios (OR) and 95% confidence intervals (CI) were calculated using multivariable logistic regression, adjusting for sex and grade level. POW: percentage of overweight

	POW at school exam (-40, 40+)				Change in POW (school exam - exam 2)			
	Unadjusted		Adjusted		Unadjusted		Adjusted	
	OR (95% CI)^a^	P^b^	OR (95% CI)^a^	P^b^	OR (95% CI)^a^	P^b^	OR (95% CI)^a^	P^b^
Boys (vs girls)	1.15 (0.70-1.88)	0.58	1.15 (0.70-1.88)	0.58	0.72 (0.44-1.18)	0.19	0.69 (0.41-1.15)	0.16
Grade		0.55		0.55		<0.01*		<0.01*
2^nd^-3^rd^	1.00 (reference)		1.00 (reference)		1.00 (reference)		1.00 (reference)	
4^th^-6^th^	0.86 (0.49-1.49)	0.59	0.87 (0.50-1.51)	0.62	1.23 (0.72-2.10)	0.45	1.19 (0.70-2.04)	0.52
7^th^-9^th^	1.20 (0.63-2.29)	0.58	1.22 (0.64-2.33)	0.55	0.34 (0.16-0.71)	<0.01*	0.32 (0.15-0.69)	<0.01*
Father BMI (kg/m2)	0.98 (0.57-1.67)	0.94	0.97 (0.57-1.67)	0.92	0.99 (0.58-1.67)	0.96	1.00 (0.58-1.72)	0.99
Mother BMI (kg/m2)	1.50 (0.83-2.70)	0.18	1.46 (0.81-2.65)	0.21	0.71 (0.38-1.30)	0.27	0.71 (0.38-1.34)	0.29
Birthweight (kg)	0.81 (0.35-1.89)	0.63	0.81 (0.34-1.91)	0.62	1.50 (0.68-3.29)	0.32	1.25 (0.50-3.15)	0.63
Hours of sleep		<0.01*		<0.01*		0.44		0.52
<8	1.00 (reference)		1.00 (reference)		1.00 (reference)		1.00 (reference)	
8	0.48 (0.25-0.92)	0.03	0.44 (0.22-0.89)	0.02*	1.55 (0.78-3.07)	0.21	0.95 (0.44-2.03)	0.89
>9	0.28 (0.13-0.60)	<0.01*	0.24 (0.10-0.56)	<0.01*	1.30 (0.61-2.75)	0.50	0.69 (0.29-1.64)	0.40
>150 weekday minutes of television (vs <150 minutes)	1.47 (1.11-1.94)	<0.01*	1.48 (1.11-1.98)	<0.01*	1.00 (0.77-1.31)	0.98	1.12 (0.84-1.49)	0.43
>150 holiday minutes of television (vs <150 minutes)	1.48 (1.05-2.08)	0.03*	1.48 (1.04-2.10)	0.03*	1.07 (0.78-1.46)	0.67	1.14 (0.83-1.58)	0.42
Physical activity	1.71 (1.01-2.89)	0.05*	1.69 (0.97-2.93)	0.06	0.81 (0.47-1.39)	0.45	0.96 (0.54-1.71)	0.89
Frequency of snacking		0.47		0.47		0.06		0.14
Every day	1.00 (reference)		1.00 (reference)		1.00 (reference)		1.00 (reference)	
Several times a week	1.22 (0.74-2.01)	0.44	1.19 (0.72-1.97)	0.50	0.57 (0.34-0.96)	0.03*	0.60 (0.36-1.03)	0.06
Do not eat	0.38 (0.04-3.33)	0.38	0.35 (0.04-3.13)	0.34	0.27 (0.03-2.39)	0.24	0.36 (0.04-3.46)	0.38

## Discussion

In this study, we accessed real-world data of school-aged children from Setagaya Ward’s annual school health checkup. We linked it to results from a sub-population of students who participated in the Obesity Health Screening program. This provided the opportunity to conduct a descriptive evaluation of schoolchildren with elevated POW at the school exam (POW >30) and to specifically examine relationships between blood test profile, lifestyle habits, and the degree of POW at the school exam and changes in POW after health screening interventions offered by the Setagaya Ward. We observed several notable trends in grade level, blood test measures, and lifestyle-related habits and their associations with POW and changes in POW.

When examining the change in POW at screening 2 of the Obesity Health Screening, we observed the lowest proportion of reductions among young elementary school children. Reduced POW was more common in 4th-6th graders and 7th-9th graders. A previous report similarly showed a decrease from school exam to screening 1 or screening 2, especially for boys in 7th-9th grade, but an increase for elementary school second-grade girls [6]. Both studies found that POW decreased more often among 7th-9th graders than among elementary school students after screening. As children get older, they may become more aware of their body shape, gain a better understanding of weight management, and develop a greater ability to maintain healthy behavior changes. Also, in 7th-9th-grade boys, POW may have improved due to height growth that coincides with puberty. The target population for the "Children's Lifestyle-related Disease Prevention Screening" conducted by the "Tokyo Health Service Association" is, in principle, the fourth grade of elementary school and the first grade of 7th-9th graders since the age at which children are considered to have the ability to make this behavior change on their own is 10 years of age or older [15]. Considering that there was little change in POW in the second grade of elementary school in this study, providing more information to parents may be necessary to optimize the outcomes of these screening efforts in the Setagaya Ward.

Among the blood tests evaluated in this study, median values of ALT, HDL-C, IRI, and UA appeared to differ by POW at school exam categories. Several measures, including AST, ALT, TC, TG, and IRI, were also associated with a change in POW. We observed a tendency toward no change in POW or an increase in POW among schoolchildren with higher AST, ALT, and TC. While this may suggest that the Obesity Health Screening has no effect, or even an adverse effect, on some schoolchildren, it is also possible that the presence or absence of illness influences this observation. Furthermore, the changes in serum cholesterol levels are consistent with previous reports [16] and are considered to be physiological changes associated with growth.

When categorizing schoolchildren by POW at the school exam, there was a significant difference in sleep time and television viewing time (both weekdays and holidays). Sleep time tended to be longer in the group with POW <40 and shorter in the group with POW >50. Some studies have suggested that sleep deficiency is associated with obesity [17-19]. Also, television viewing time on weekdays and holidays tended to be lower in the group with POW <40 and higher in the group with POW >50. The relationship between television viewing time and obesity has been reported previously [20,21], and the results of this study are consistent with the idea that extended viewing time offsets the time needed for physical activity and is also associated with other unhealthy behaviors. To improve Obesity Health Screening efforts, it may be essential to identify new strategies during counseling and guidance provided during health examinations to encourage schoolchildren and parents to improve lifestyle habits. For example, it may be effective to distribute information about obesity when notifying children of their upcoming medical checkups. One idea would be to provide detailed information about the risks and remedial measures related to obesity, not only to children but also to their parents, using pamphlets, videos, and other media.

A significant limitation to consider is the incomplete application (screening 1) rate and consultation (screening 2) rate for the Obesity Health Screening. The application rate among those eligible for the screening was about 61-73%, and the consultation rate was about 57-66% from 2015 to 2019. For both screenings 1 and 2, there were a few candidate dates, each limited to three days. This likely resulted in a significant decrease in attendance, as the subjects' schedules did not align with or overlap with school events. While this proportion represents the majority, a subset of the population not included in this study may have unique characteristics. This decrease may reflect results among subjects who are more health-conscious, as it could be due to factors such as low health literacy, fewer resources to improve lifestyles, or low motivation to take action.

Furthermore, missing data on lifestyle habits, including parental characteristics, pertain to participants who did not submit the dietary questionnaire used for nutritional guidance, i.e., participants who did not wish to receive nutritional guidance. We cannot determine whether participants did not want to receive nutritional guidance or whether the schedule did not suit them. Still, because participants' motivation to improve their obesity influences their decision to participate, we cannot rule out bias.

Additionally, there were very few girls in the POW >50 category, which may affect the interpretation of the gender-stratified analyses. Furthermore, although many outcomes were tested independently during the analysis, factors with significant associations may be identified by chance, so we believe the results should be interpreted with caution. The current study population may be over-representing individuals who are relatively motivated to improve their obesity status and/or those from a specific socioeconomic demographic. In other words, rural and low-resource settings may have higher rates of obesity among children than the study area. Still, they may also have lower health literacy and fewer resources to improve their lifestyles. Whether a screening program model is feasible and effective in such settings remains to be seen. Nevertheless, we consider it useful to examine real-world circumstances among schoolchildren who tend to participate in such voluntary Obesity Health Screening programs. The incidence of obese children in the target area of this study is low compared to Japan as a whole [6], so it is difficult to say whether these results are generalizable to Japan as a whole. Still, it is likely reflective of the situation in urban areas. Finally, this study did not design and conduct screening as a research study; its primary purpose was to examine whether there was room for improvement in the screening carried out by local governments over many years. Therefore, from a scientific perspective, there are various problems. Limitations, including the lack of a control group, a short follow-up period, and significant selection bias due to incomplete participation, affect the results, so we believe that great care must be taken when interpreting them.

## Conclusions

In this unique opportunity to examine data from the real-world context of the Obesity Health Screening program in Setagaya Ward over a recent five-year period, we provided descriptive results on POW status and improvements by various child and parental characteristics, blood test measurements, and lifestyle factors. Results suggest greater improvements among older children, gender differences, and blood test and lifestyle profiles consistent with previous reports. It may be essential to more fully integrate effective guidance on healthy lifestyle habits and to extend the program to include careful follow-up for schoolchildren with abnormalities such as liver dysfunction and dyslipidemia, which can benefit both clinical outcomes and provide additional research prospects in the future.
